# Acute metabolic responses to a high-carbohydrate meal in outpatients with type 2 diabetes treated with a low-carbohydrate diet: a crossover meal tolerance study

**DOI:** 10.1186/1743-7075-6-52

**Published:** 2009-12-29

**Authors:** Hajime Haimoto, Tae Sasakabe, Hiroyuki Umegaki, Kenji Wakai

**Affiliations:** 1 Department of Internal Medicine, Haimoto Clinic, 1-80 Yayoi, Kasugai, Aichi 486-0838, Japan; 2 Department of Clinical Nutrition, Haimoto Clinic, 1-80 Yayoi, Kasugai, Aichi 486-0838, Japan; 3 Department of Geriatrics, Nagoya University Graduate School of Medicine, 65 Tsurumai, Showa, Nagoya, Aichi 466-8550, Japan; 4 Department of Preventive Medicine/Biostatistics and Medical Decision Making, Nagoya University Graduate School of Medicine, 65 Tsurumai, Showa, Nagoya, Aichi 466-8550, Japan

## Abstract

**Background:**

A low-carbohydrate diet (LCD) achieves good glycemic control in type 2 diabetes (T2DM) compared with a high-carbohydrate diet. With respect to energy metabolism, acute metabolic responses to high-carbohydrate meals (HCMs) have not been determined in LCD patients with T2DM.

**Subjects and methods:**

We enrolled 31 subjects with T2DM (mean age: 62 yrs, mean hemoglobin A1c level: 6.9%), of whom 13 were on a strict LCD (26% carbohydrate diet), and 18 a moderate one (44% carbohydrate diet). Two isocaloric meals were administered to all subjects in a randomized crossover design. The carbohydrate:protein:fat ratios of HCMs and low-carbohydrate meals (LCMs) were 59:20:21 and 7:20:73, respectively. Serum β-hydroxybutyrate, acetoacetate, free fatty acids (FFAs), triglyceride and insulin, and plasma glucose concentrations were measured for 120 minutes after the intake of each meal.

**Results:**

HCMs rapidly decreased postprandial β-hydroxybutyrate, acetoacetate and FFA concentrations within 2 hours in all patients in combination with rapid increases in serum insulin and plasma glucose, while LCMs increased or did not change β-hydroxybutyrate, acetoacetate and FFAs (*P *< 0.001 for all). HCMs did not change postprandial triglyceride concentrations over 2 hours, while LCMs gradually increased them (*P *< 0.001).

HCMs sharply and rapidly decreased postprandial β-hydroxybutyrate and acetoacetate concentrations in strict LCD subjects over 2 hours, but only slightly decreased them in moderate LCD subjects (*P *< 0.001, difference between strict and moderate LCD subjects). The parameter Δketone bodies (level at 120 minutes - level at baseline) was significantly correlated with the insulinogenic index (Spearman's r = 0.503 for β-hydroxybutyrate and 0.509 for acetoacetate), but not with total insulin secretory capacity. Moreover, HCMs slightly decreased postprandial triglyceride levels in strict LCD subjects but somewhat increased them in the moderate LCD subjects (*P *= 0.002). The parameter Δtriglyceride was significantly correlated with background dietary %carbohydrate (Spearman's r = 0.484).

**Conclusion:**

HCMs rapidly decreased postprandial ketone body concentrations in T2DM patients treated with a LCD. The decreases were more remarkable in strict than in moderate LCD subjects. HCMs slightly decreased postprandial triglyceride levels in strict LCD subjects. The parameter Δketone bodies was significantly correlated with the insulinogenic index, as was Δtriglyceride with background dietary %carbohydrate.

## Introduction

Low-carbohydrate diets (LCDs) are known to be more effective than high-carbohydrate diets (HCDs) in improving glycemic control in type 2 diabetes mellitus (T2DM) [1,2]. Our carbohydrate-reduced diet (CARD) also showed efficacy and safety over 2 years in mild T2DM [3], with an efficacy comparable to that provided by insulin therapy in severe T2DM [4]. An important problem with LCDs, however, is the high attrition rate [2,3,5]. Even patients who strictly conform to LCDs sometimes crave a high-carbohydrate meal (HCM), which often triggers the patients to drop out from the LCDs.

Although a key adaptation of LCDs is the production and utilization of ketone bodies [6,7], the potential of such bodies for uses other than as fuels remains unclear [7]. Even moderate LCDs (30-45% carbohydrate diets) are highly effective for T2DM and dyslipidemia [2,3,8]. Thus, patients on a less-strict LCD sometimes eat HCMs because they are not required to strictly limit carbohydrates. Nevertheless, little is known about the acute metabolic changes resulting from the change in carbohydrate conditions in such patients.

In the present study, our subjects, T2DM outpatients on CARD, underwent crossover meal tolerance tests (HCM vs. a low-carbohydrate meal, LCM) to compare the acute effects of HCMs and LCMs on postprandial serum ketone bodies, free fatty acids (FFAs) and triglycerides. We also compared acute responses between subjects on moderate (non-ketogenic) and strict (ketogenic) CARD. Furthermore, for HCMs, we examined the correlation of postprandial changes in serum ketone bodies, FFAs and triglycerides with an increase in postprandial plasma glucose, insulin secretory capacity and background dietary %carbohydrate.

## Subjects and methods

Thirty-one volunteer T2DM outpatients from the Haimoto Clinic participated in the study (Table 1). All patients were Japanese. Patients with severe diabetes complications were excluded. The participants achieved relatively good glycemic control (mean hemoglobin A1c (HbA1c) level: 6.9 ± 0.4%) by implementing CARD for a mean duration of 22 months. They received no insulin, sulfonylurea or nateglinide treatment, and their mean HbA1c level before CARD was 8.9 ± 2.0%. Based on their initial HbA1c levels, 18 were assigned moderate CARD and 13 strict CARD [3,4]. Nine subjects took glucose-lowering medication (miglitol, metformin and pioglitazone).

**Table 1:** Background characteristics of participants

	All subjects (n = 31)	Moderate CARD subjects (n = 18)	Strict CARD subjects (n = 13)	P*
Male/Female	17/14	9/9	8/5	
Age (yrs)	62 ± 7	62 ± 6	62 ± 7	0.98
Body mass index	23.2 ± 3.4	22.9 ± 3.3	23.6 ± 3.5	0.56
Duration of diabetes (months)	89 ± 55	80 ± 40	101 ± 68	0.34
Duration of CARD (months)	22 ± 16	27 ± 16	16 ± 13	0.040
HbA1c (%)	6.9 ± 0.4	6.9 ± 0.4	6.8 ± 0.4	0.37
HbA1c before CARD (%)	8.9 ± 2.0	8.3 ± 1.4	9.6 ± 2.4	0.099
Serum LDL cholesterol (mg/dl)	117 ± 21	110 ± 17	126 ± 22	0.040
Serum HDL cholesterol (mg/dl)	62 ± 12	61 ± 13	64 ± 10	0.57
Serum triglyceride (mg/dl)	94 ± 42	98 ± 37	111 ± 58	0.47
Dietary intake				
Total energy (kcal/day)	1808 ± 495	1713 ± 423	1990 ± 571	0.15
Carbohydrate (g/day)	158 ± 48	179 ± 35	127 ± 46	0.002
Carbohydrate (% energy)	36 ± 10	42 ± 4	26 ± 7	< 0.001
Fat (g/day)	75 ± 21	67 ± 17	92 ± 26	0.006
Fat (% energy)	38 ± 9	35 ± 5	44 ± 12	0.033
Protein (g/day)	81 ± 21	78 ± 22	90 ± 21	0.13
Protein (% energy)	18 ± 3	18 ± 3	19 ± 4	0.71

Plus-minus values are means ± SD. CARD: Carbohydrate-reduced diet, HbA1c: Hemoglobin A1c.

*: *P *for the difference between the moderate and strict CARD subjects.

The HCMs and LCMs were prepared at the Haimoto Clinic by an experienced dietitian. The total energy of the two meals was identical, while the macronutrient composition was contrastive (Table 2). The target HCM macronutrient composition followed the guidelines of the Japan Diabetes Society [9]. The background dietary intake of the subjects was assessed using 3-day food records.

**Table 2:** Composition of test meals

	**Low-carbohydrate meal**	**High-carbohydrate meal**
Total energy (kcal)	500	500
Carbohydrate (% energy)	7	59
Fat (% energy)	73	21
Protein (% energy)	20	20
Carbohydrate (g)	9	73
Fat (g)	42	12
Saturated fatty acids (g)	12	3
Monounsaturated fatty acids (g)	17	3
Polyunsaturated fatty acids (g)	9	3
Protein (g)	23	23
Menu	Pork back rib char boiled (86 g), tuna salad (59 g), mixed beans (20 g), lettuce (10 g), boiled egg (50 g), tomato (10 g)	Rice cake (140 g), mackerel boiled Japanese bean taste (36 g), beans and seaweeds (33 g), cooked egg (50 g), lettuce (10 g), tofu (soybean curd) soup (33 g), tomato (10 g), apple (20 g)

All 31 participants underwent two-meal tolerance tests at intervals of 7-14 days. They were randomly assigned to two groups: 15 subjects took HCMs and LCMs in the first and second tests, respectively, while 16 subjects received LCMs and HCMs in the first and second sessions, respectively. Each meal test was conducted after a 12-hour overnight fast. The subjects were asked to continue their CARD and physical activity during the study period. After blood sampling at baseline (0 minutes), the subjects were asked to consume the test meal in 10 minutes, and blood was collected after 30, 60 and 120 minutes. All antidiabetic and lipid-lowering drugs were stopped for 24 hours before each tolerance test.

Plasma glucose concentration was determined by enzymatic methods (Shino-Test Co. Kanagawa, Japan). Serum immunoreactive insulin was measured using the standard double antibody radioimmunoassay method (Fujirebio Inc. Tokyo, Japan). Enzymatic methods were used to measure serum total cholesterol (Sysmex Co., Hyogo, Japan), triglycerides (Daiichi Pure Chemicals Co., Tokyo, Japan), free fatty acids (Eiken Chemicals Co., Tokyo, Japan), β-hydroxybutyrate and acetoacetate (Kainos Laboratories Inc., Tokyo, Japan). Direct methods were used to assay serum LDL-cholesterol and HDL-cholesterol (Daiichi Pure Chemicals Co., Tokyo, Japan). HbA1c level was measured by high-performance liquid chromatography (Arkley Co., Kyoto, Japan).

The study protocol was approved by the Ethical Committee of the Nagoya Tokusyukai General Hospital, and all participants provided written informed consent.

### Statistical analysis

Repeated-measures analysis of variance (ANOVA) was used to examine differences in the time course of each substance. The baseline levels of each biomarker were compared between the two meals by paired *t*-test, and those between the moderate and strict CARD groups were compared by unpaired *t*-test.

With respect to the responses to HCMs, we computed Spearman's correlation coefficients to examine the correlation of the changes in serum postprandial ketone body (Δβ-hydroxybutyrate and Δacetoacetate), FFA (ΔFFAs) and triglyceride (Δtriglyceride) levels with an increase in plasma glucose levels, insulin secretory capacity and background dietary %carbohydrate as assessed by 3-day food records. The parameters Δketone bodies, ΔFFAs and Δtriglyceride were defined as the level at 120 minutes minus the level at baseline. The incremental plasma glucose (Δglucose) and serum insulin (Δinsulin) were calculated as the difference between the baseline and the peak. The trapezoidal rule was used to calculate the incremental area under the curve for glucose (AUC-glucose) and insulin (AUC-insulin). The HOMA-R and insulinogenic index were computed as follows: [fasting plasma glucose (mg/dl)] × [fasting serum insulin (μIU/ml)]/405 and ([serum insulin at 30 minutes] - [serum insulin at baseline])/([plasma glucose at 30 minutes] - [plasma glucose at baseline]), respectively (9).

All values were expressed as means ± SD. *P *< 0.05 was considered statistically significant.

## Results

### Comparison between HCM and LCM

**Figure 1:**
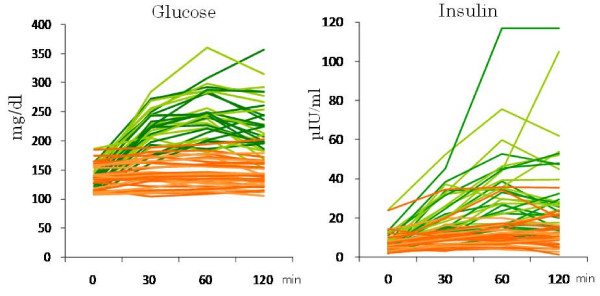
**Postprandial glucose and insulin concentrations under HCM (green) and LCM (orange) in all individual subjects**. HCMs caused postprandial glucose and insulin concentrations to increase rapidly, while LCMs increased them only slightly (*P *< 0.001 for the difference between the two meals for both glucose and insulin).

**Figure 2:**
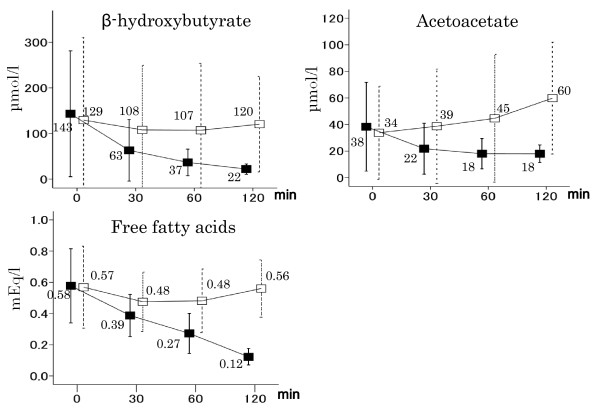
**Postprandial β-hydroxybutyrate, acetoacetate and FFA concentrations under HCM and LCM in all subjects**. Vertical lines show means ± SD. HCMs (black square) rapidly decreased postprandial β-hydroxybutyrate, acetoacetate and FFA concentrations, while LCMs (white square) increased or did not change them (*P *< 0.001 for the difference between the two meals for β-hydroxybutyrate, acetoacetate and FFAs).

**Figure 3:**
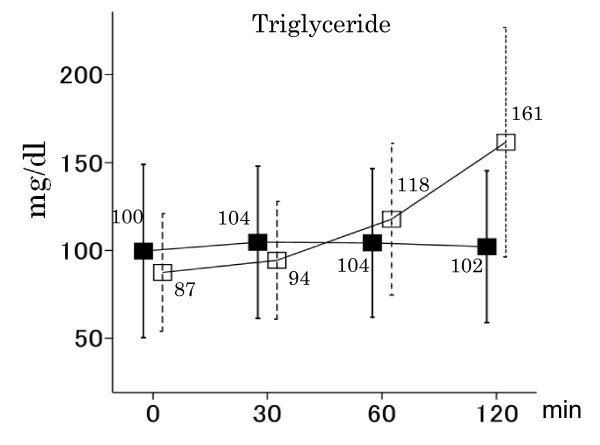
**Postprandial triglyceride concentrations under HCM and LCM in all subjects**. Vertical lines show means ± SD. LCMs (white square) increased postprandial triglyceride concentrations, while HCMs (black square) induced little change (*P *< 0.001 for the difference between the two meals).

The baseline concentrations of all substances were not significantly different between the HCM and LCM tolerance tests (Figures 1, 2 and 3). LCMs increased postprandial glucose and insulin concentrations only slightly (139 ± 21 mg/dl and 6.4 ± 4.6 μIU/ml at baseline, 143 ± 23 mg/dl and 10.3 ± 6.4 μIU/ml after 30 min, 144 ± 23 mg/dl and 11.6 ± 7.4 μIU/ml after 60 min, and 142 ± 22 mg/dl and 11.4 ± 7.4 μIU/ml after 120 min, respectively), while HCMs caused them to increase rapidly (134 ± 20 mg/dl and 6.6 ± 4.7 μIU/ml at baseline, 223 ± 33 mg/dl and 21.7 ± 1.5 μIU/ml after 30 min, 245 ± 42 mg/dl and 33.6 ± 21.8 μIU/ml after 60 min, and 227 ± 49 mg/dl and 34.6 ± 24.9 μIU/ml after 120 min, respectively) (*P *< 0.001 for the difference between the two meals for both glucose and insulin; Figure 1). HCMs rapidly decreased postprandial β-hydroxybutyrate, acetoacetate and FFA concentrations, while LCMs increased or did not change them (*P *< 0.001 for the difference between the two meals for all substances; Figure 2). LCMs increased postprandial triglyceride concentrations, while HCMs induced little change (*P *< 0.001 for the difference between the two meals; Figure 3).

### Background characteristics of the moderate and strict CARD groups

Background total energy intake was not significantly different between the two groups (Table 1). The daily average intakes of carbohydrates and fat, however, differed greatly, with the strict and moderate CARD groups achieving 26% and 42% carbohydrate diets, respectively. The baseline concentrations of serum β-hydroxybutyrate and acetoacetate were significantly higher in the strict CARD group before HCM and LCM (*P *< 0.02 for all; Table 3). Those of other substances were not significantly different between the two groups, with the exception that the strict CARD group showed a higher serum FFA level before the LCM test (*P *= 0.04).

**Table 3:** Differences in the time course for postprandial plasma glucose and serum insulin, ketone body, FFA and triglyceride levels between the moderate and strict CARD subjects after intake of the two meals

	High-carbohydrate meal								
	Moderate CARD subjects (n = 18)				Strict CARD subjects (n = 13)				P*
	**Baseline**	**30 min**	**60 min**	**120 min**	**Baseline**	**30 min**	**60 min**	**120 min**	
Glucose (mg/dl)	129 ± 18	214 ± 34	229 ± 35	213 ± 39	142 ± 20	223 ± 33	245 ± 42	227 ± 48	0.13
Insulin (μIU/ml)	6.2 ± 4.6	24.2 ± 13.4	36.3 ± 26.4	37.3 ± 30.8	6.2 ± 2.2	18.2 ± 7.3	29.9 ± 13.2	30.8 ± 13.6	0.51
BHB (μmol/l)	80 ± 55	33 ± 14	25 ± 7	17 ± 4	231 ± 169	104 ± 89	53 ± 39	29 ± 14	0.001
Acetoacetate(μmol/l)	22.5 ± 13.8	13.8 ± 5.3	13.5 ± 5.6	15.4 ± 4.1	60.2 ± 40.5	32.9 ± 25.4	24.3 ± 14.3	21.5 ± 7.9	0.001
FFAs (mEq/l)	0.50 ± 0.15	0.35 ± 0.13	0.24 ± 0.10	0.11 ± 0.04	0.69 ± 0.29	0.44 ± 0.14	0.31 ± 0.16	0.14 ± 0.07	0.06
Triglyceride (mg/dl)	94 ± 41	101 ± 41	103 ± 40	107 ± 43	108 ± 59	109 ± 48	106 ± 47	95 ± 44	0.014

	**Low-carbohydrate meal**								
	**Moderate CARD subjects (n = 18)**				**Strict CARD subjects (n = 13)**				P*

	**Baseline**	**30 min**	**60 min**	**120 min**	**Baseline**	**30 min**	**60 min**	**120 min**	
Glucose (mg/dl)	135 ± 23	137 ± 25	139 ± 25	137 ± 25	144 ± 19	151 ± 16	151 ± 19	149 ± 16	0.35
Insulin (μIU/ml)	7.1 ± 5.7	10.9 ± 7.8	12.7 ± 9.3	11.7 ± 8.8	5.5 ± 2.4	9.6 ± 3.3	10.1 ± 3.1	10.9 ± 5.2	0.67
BHB (μmol/l)	56 ± 30	51 ± 19	59 ± 23	78 ± 32	232 ± 247	187 ± 195	174 ± 211	179 ± 139	0.012
Acetoacetate (μmol/l)	17.4 ± 9.1	20.6 ± 8.0	27.1 ± 11.0	42.1 ± 22.0	56.5 ± 44.6	63.9 ± 57.9	69.0 ± 67.1	84.4 ± 51.2	0.95
FFAs (mEq/l)	0.52 ± 0.21	0.46 ± 0.22	0.46 ± 0.22	0.54 ± 0.21	0.63 ± 0.32	0.50 ± 0.15	0.52 ± 0.19	0.59 ± 0.14	0.53
Triglyceride (mg/dl)	95 ± 35	103 ± 36	125 ± 44	169 ± 62	77 ± 30	83 ± 27	108 ± 42	152 ± 71	0.89

Values are means ± SD. CARD: carbohydrate-reduced diet, BHB: β-hydroxybutyrate, FFAs: free fatty acids.

*: *P *for the difference in the time course between the moderate and strict CARD subjects.

### Differences between the moderate and strict CARDs in two-meal tests

No significant differences were found in the time courses of the postprandial glucose or insulin levels between the moderate and strict CARD groups after the intake of HCM and LCM (*P *> 0.05 for all; Table 3).

**Figure 4:**
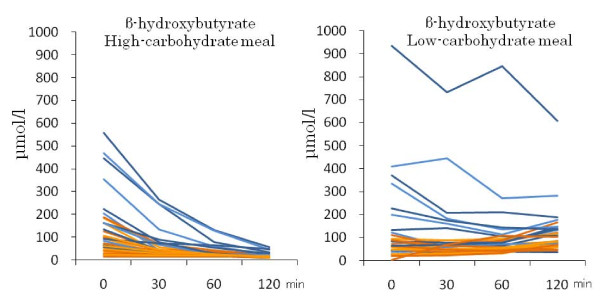
**Individual changes in postprandial β-hydroxybutyrate levels in the moderate (orange) and strict (blue) CARD subjects after the intake of HCM and LCM**.

**Figure 5:**
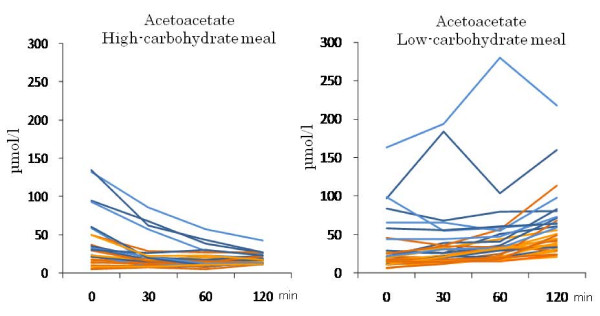
**Individual changes in postprandial acetoacetate levels in the moderate (orange) and strict (blue) CARD subjects after the intake of HCM and LCM**.

HCMs sharply decreased the postprandial serum β-hydroxybutyrate and acetoacetate concentrations in the strict CARD group, and only slightly decreased them in the moderate group (Table 3, Figures 4 and 5). The time courses were significantly different between the two groups (*P *= 0.001 for both). LCMs slightly decreased the postprandial β-hydroxybutyrate levels in the strict CARD group, and increased them somewhat in the moderate group (*P *= 0.012 for the difference in the time course; Table 3 and Figure 4). LCMs gradually increased the postprandial acetoacetate concentrations in both groups (*P *= 0.95 for the difference in the time course; Table 3 and Figure 5). The time course of postprandial FFA concentrations was not significantly different between the two groups for both meals.

**Figure 6:**
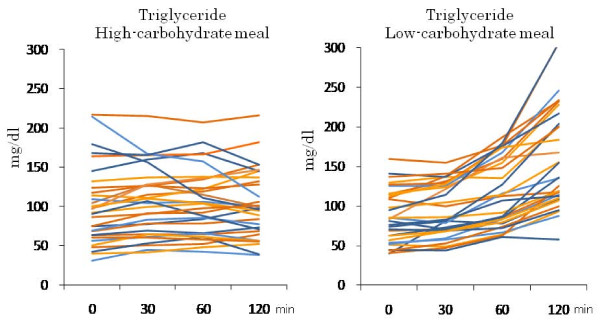
**Individual changes in postprandial triglyceride levels in the moderate (orange) and strict (blue) CARD subjects after the intake of HCM and LCM**.

HCMs slightly decreased postprandial triglyceride levels in the strict CARD group over 120 minutes, and slightly increased them in the moderate group (Table 3 and Figure 6). The time courses of the two groups were significantly different (*P *= 0.014). LCMs increased the levels linearly over 120 minutes in both groups (*P *= 0.89 for the difference in the time course).

### Correlation of changes in postprandial serum levels of ketone bodies, FFAs and triglycerides with insulin secretory capacity, background dietary %carbohydrate and increases in glucose levels after intake of HCM

**Figure 7:**
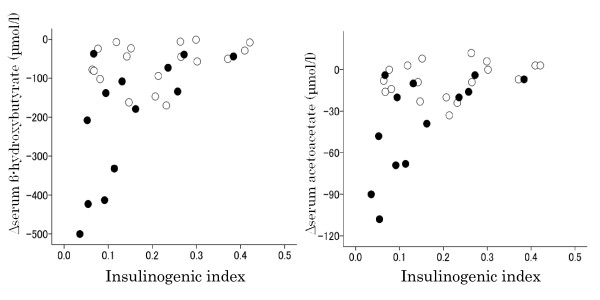
**The relationship between Δketone bodies (level at 120 minutes - level at baseline) and the insulinogenic index after the intake of HCM**. Closed and open circles indicate subjects in the strict and moderate CARD groups, respectively. The parameter Δketone bodies was significantly correlated with the insulinogenic index (β-hydroxybutyrate: Spearman's r = 0.503, *P *= 0.004; acetoacetate: Spearman's r = 0.509, *P *= 0.003).

**Figure 8:**
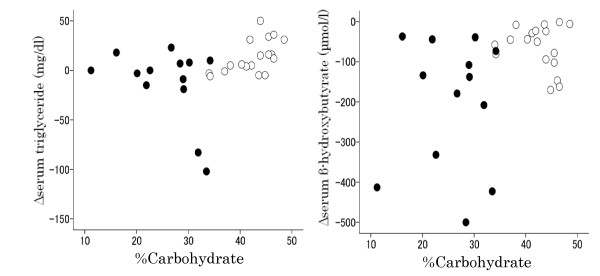
**The relationship between Δtriglyceride or Δβ-hydroxybutyrate (level at 120 minutes - level at baseline) and background dietary %carbohydrate after the intake of HCM**. Closed and open circles indicate subjects in the strict and moderate CARD groups, respectively. Background dietary %carbohydrate was strongly correlated with Δtriglyceride (Spearman's r = 0.484, *P *= 0.006) and was moderately correlated with Δβ-hydroxybutyrate (Spearman's r = 0.380, *P *= 0.035). Excluding the two subjects with a greatest decrease of serum triglycerides did not materially alter the association between Δtriglyceride and dietary %carbohydrate (Spearman's r = 0.487 [P = 0.007]).

Both Δβ-hydroxybutyrate and Δacetoacetate were strongly and positively correlated with the insulinogenic index (Table 4 and Figure 7), moderately and inversely with Δglucose and AUC-glucose and moderately and positively with background dietary %carbohydrate (Table 4 and Figure 8), while they were not significantly correlated with fasting plasma glucose, fasting serum insulin, Δinsulin, AUC-insulin and HOMA-R. Namely, postprandial serum ketone body levels decreased more in subjects with lower insulinogenic indices. The results for Δ FFAs were similar to those for Δketone bodies, but the correlation was somewhat weaker.

**Table 4:** Correlation of changes in serum ketone bodies, FFAs and triglycerides with insulin response capacity, background dietary %carbohydrate and increases in plasma glucose after intake of the high-carbohydrate meal

	Δβ-hydroxybutyrate	Δacetoacetate	ΔFFAs	Δtriglyceride
Fasting plasma glucose	-0.261 (0.156)	-0.208 (0.26)	-0.208 (0.26)	0.001 (0.10)
Δglucose	-0.472 (0.007)	-0.442 (0.012)	-0.302 (0.10)	-0.250 (0.18)
AUC-glucose	-0.450 (0.011)	-0.407 (0.023)	-0.358 (0.048)	-0.149 (0.42)
Fasting serum insulin	0.279 (0.13)	0.145 (0.44)	-0.114 (0.54)	-0.146 (0.42)
Δinsulin	0.328 (0.072)	0.268 (0.15)	0.012 (0.95)	-0.097 (0.60)
AUC-insulin	0.346 (0.057)	0.265 (0.15)	-0.007 (0.97)	-0.165 (0.38)
Insulinogenic index	0.503 (0.004)	0.509 (0.003)	0.359 (0.047)	0.048 (0.80)
HOMA-R	0.195 (0.292)	-0.129 (0.488)	0.035 (0.85)	-0.238 (0.20)
%Carbohydrate	0.380 (0.035)	0.372 (0.040)	0.268 (0.145)	0.484 (0.006)

Values indicate Spearman's r with *P *values in parentheses. FFAs: free fatty acids, AUC: area under curve.

Δglucose and Δinsulin: level at the peak - level at baseline.

Δβ-hydroxybutyrate, Δacetoacetate, Δfree fatty acids (ΔFFAs) and Δtriglyceride: level at 120 minutes - level at baseline.

Background dietary %carbohydrate was strongly and positively correlated with Δ triglyceride and was moderately correlated with Δβ-hydroxybutyrate and Δ acetoacetate. The parameter Δ triglyceride was not significantly correlated with any variables other than background dietary %carbohydrate (Table 4 and Figure 8). This means that postprandial serum triglycerides decreased more in subjects with lower background dietary %carbohydrate. Excluding the two subjects with a greatest decrease of serum triglycerides did not materially alter the association between Δtriglyceride and dietary %carbohydrate (Spearman's r = 0.487 [P = 0.007]).

In terms of the baseline levels of ketone bodies, the insulinogenic index was significantly and inversely correlated with baseline β-hydroxybutyrate and acetoacetate levels (Spearman's r = -0.523 [*P *= 0.003] and -0.513 [*P *= 0.003], respectively), as was dietary %carbohydrate (Spearman's r = -0.421 [P = 0.018] and -0.443 [*P *= 0.013], respectively).

## Discussion

The limitations of the present study are its very short term and the large SD in serum ketone body levels before the meal tests. Large between-person variations in fasting serum β-hydroxybutyrate levels have been previously reported in outpatients after long-term LCD treatment [10,11]. Another limitation is that the use of glucose and lipid-lowering drugs may have affected the results.

Nevertheless, the present study demonstrated that HCMs including 59% carbohydrates rapidly decreased postprandial serum ketone body and FFA concentrations within 2 hours in T2DM outpatients on CARD. The decrease can be explained in terms of the metabolic changes of ketone bodies and FFAs to glucose to be used as fuel [12], which is well-known to be largely determined by insulin secretion after the intake of large amounts of carbohydrates [12,13]. However, our study clarified that the decrease in serum ketone body levels was much more rapid than expected from starvation conditions [12,14] and was more remarkable in strict (ketogenic) CARD subjects than in moderate (non-ketogenic) CARD subjects. Carbohydrate or glucose administration to fasting normals causes serum ketone body levels to decrease in a few days [12,14]. Under low-carbohydrate conditions, unlike under starvation conditions, a sufficient intake of protein and fat is likely to be involved in the rapid decrease of serum ketone body levels.

The present study also demonstrated that the insulinogenic index was strongly and positively correlated with Δketone bodies while concomitantly showing a strong and inverse correlation with baseline ketone body levels, but total insulin secretory capacity (AUC-insulin) was not significantly correlated with Δketone bodies. A possible interpretation of these findings is that under low-carbohydrate conditions, a lower insulinogenic index is probably a factor in maintaining a high level of baseline ketone bodies independent of dietary %carbohydrate; then, the initial secretion of only a small amount of insulin after the intake of LCMs leads to rapid decreases of serum postprandial ketone body levels.

Postprandial triglyceride levels have attracted much attention as a cardiovascular risk factor [15,16], and LCDs are well known to improve serum triglyceride profiles compared to HCDs [7,13,17]. Small amounts of dietary fats (15 g) slightly increase postprandial serum triglyceride levels, but never decrease them in normolipidemic adults [18]. Interestingly, the present study demonstrated that small amounts of fat (12 g) in HCMs slightly decreased these levels over 2 hours in the strict CARD group in contrast to causing a slight increase in the moderate group. Moreover, Δtriglyceride was strongly and positively correlated with background dietary %carbohydrate, but not with an increase in postprandial plasma glucose and serum insulin, insulinogenic index or total insulin secretory capacity. Specifically, postprandial triglyceride levels decreased more in strict CARD subjects with lower %carbohydrate levels, and such patients had higher baseline ketone body levels. Given that ketone bodies provide the same metabolic effects as insulin and inhibit lipolysis [7,19,20], serum ketone bodies may play a role in decreasing postprandial triglyceride levels. In contrast, large amounts of fat (42 g) in LCMs increased triglyceride concentrations in both CARD groups. These results raise the possibility of a threshold in the dietary fat level under which postprandial triglyceride levels are not elevated [21].

In summary, when T2DM patients treated with CARD ingested HCMs, rapid metabolic change was induced within 2 hours; a rapid decrease in serum ketone bodies and FFA levels was found in conjunction with an increase in postprandial glucose and insulin levels. The decrease in ketone bodies was more remarkable in strict than in moderate CARD subjects. The parameter Δketone bodies was significantly correlated with the insulinogenic index, but not with total insulin secetory capacity. HCMs gradually decreased postprandial triglyceride levels in strict CARD subjects in contrast to causing a slight increase in moderate CARD subjects. The parameter Δtriglyceride was significantly correlated with dietary %carbohydrate.

## Abbreviations

CARD: carbohydrate-reduced diet; FFAs: free fatty acids; HbA1c: hemoglobin A1c; HCD: high-carbohydrate diet; HCM: high-carbohydrate meal; LCD: low-carbohydrate diet; LCM: low-carbohydrate meal; T2DM: type 2 diabetes mellitus.

## Competing interests

The authors declare that they have no competing interests.

## Authors' contributions

HH and TS designed the study and participated in data collection. HH, KW and HU performed statistical analysis and interpretation. HH and KW wrote the manuscript. All authors read and approved the final manuscript.

## References

[B1] Accurso A Bernstein RK Dahlqvist A Draznin B Feinman RD Fine EJ Gleed A Jacobs DB Larson G Lustig RH Manninen AH McFarlane SI Morrison K Nielsen JV Ravnskov U Roth KS Silvestre R Sowers JR Sundberg R Volek JS Westman EC Wood RJ Wortman J Vernon MC Dietary carbohydrate restriction in type 2 diabetes mellitus and metabolic syndrome: time for a critical appraisal Nutr Metab (Lond) 2008 5 9 10.1186/1743-7075-5-9 18397522 PMC2359752

[B2] Westman EC Yancy WS Jr Mavropoulos JC Marquart M McDuffie JR The effect of a low-carbohydrate, ketogenic diet versus a low-glycemic index diet on glycemic control in type 2 diabetes mellitus Nutr Metab (Lond) 2008 5 36 10.1186/1743-7075-5-36 19099589 PMC2633336

[B3] Haimoto H Iwata M Wakai K Umegaki H Long-term effects of a diet loosely restricting carbohydrates on HbA1c levels, BMI and tapering of sulfonylureas in type 2 diabetes: a 2-year follow-up study Diabetes Res Clin Pract 2008 79 350 356 10.1016/j.diabres.2007.09.009 17980451

[B4] Haimoto H Sasakabe T Wakai K Umegaki H Effects of a low-carbohydrate diet on glycemic control in outpatients with severe type 2 diabetes Nutr Metab (Lond) 2009 6 21 10.1186/1743-7075-6-21 19419563 PMC2690585

[B5] Daly ME Paisey R Millward BA Eccles C Williams K Hammersley S MacLeod KM Gale TJ Short-term effects of severe dietary carbohydrate-restriction advice in Type 2 diabetes--a randomized controlled trial Diabet Med 2006 23 15 20 10.1111/j.1464-5491.2005.01760.x 16409560

[B6] Westman EC Feinman RD Mavropoulos JC Vernon MC Volek JS Wortman JA Yancy WS Phinney SD Low-carbohydrate nutrition and metabolism Am J Clin Nutr 2007 86 276 284 17684196 10.1093/ajcn/86.2.276

[B7] Volek JS Phinney SD Forsythe CE Quann EE Wood RJ Puglisi MJ Kraemer WJ Bibus DM Fernandez ML Feinman RD Carbohydrate restriction has a more favorable impact on the metabolic syndrome than a low fat diet Lipids 2009 44 297 309 10.1007/s11745-008-3274-2 19082851

[B8] Johnston CS Tjonn SL Swan PD White A Hutchins H Sears B Ketogenic low-carbohydrate diets have no metabolic advantage over nonketogenic low-carbohydrate diets Am J Clin Nutr 2006 83 1055 1061 16685046 10.1093/ajcn/83.5.1055

[B9] Japan Diabetes Society Treatment Guide for Diabetes Bunkodo 2007

[B10] Noakes M Foster PR Keogh JB James AP Mamo JC Clifton PM Comparison of isocaloric very low carbohydrate/high saturated fat and high carbohydrate/low saturated fat diets on body composition and cardiovascular risk Nutr Metab (Lond) 2006 3 7 10.1186/1743-7075-3-7 16403234 PMC1368980

[B11] Dyson PA Beatty S Matthews DR A low-carbohydrate diet is more effective in reducing body weight than healthy eating in both diabetic and non-diabetic subjects Diabet Med 2007 24 1430 1435 10.1111/j.1464-5491.2007.02290.x 17971178

[B12] Cahill GF Jr Fuel metabolism in starvation Annu Rev Nutr 2006 26 1 22 10.1146/annurev.nutr.26.061505.111258 16848698

[B13] Volek JS Fernandez ML Feinman RD Phinney SD Dietary carbohydrate restriction induces a unique metabolic state positively affecting atherogenic dyslipidemia, fatty acid partitioning, and metabolic syndrome Prog Lipid Res 2008 47 307 318 10.1016/j.plipres.2008.02.003 18396172

[B14] Aoki TT Muller WA Brennan MF Cahill GF Jr Metabolic effects of glucose in brief and prolonged fasted man Am J Clin Nutr 1975 28 507 511 1130310 10.1093/ajcn/28.5.507

[B15] Eberly LE Stamler J Neaton JD Relation of triglyceride levels, fasting and nonfasting, to fatal and nonfatal coronary heart disease Arch Intern Med 2003 163 1077 1083 10.1001/archinte.163.9.1077 12742806

[B16] O'Keefe JH Bell DS Postprandial hyperglycemia/hyperlipidemia (postprandial dysmetabolism) is a cardiovascular risk factor Am J Cardiol 2007 100 899 904 10.1016/j.amjcard.2007.03.107 17719342

[B17] Katan MB Alternatives to low-fat diets Am J Clin Nutr 2006 83 989 990 16685038 10.1093/ajcn/83.5.989

[B18] Dubois C Beaumier G Juhel C Armand M Portugal H Pauli AM Borel P Latge C Lairon D Effects of graded amounts (0-50 g) of dietary fat on postprandial lipemia and lipoproteins in normolipidemic adults Am J Clin Nutr 1998 67 31 38 9440372 10.1093/ajcn/67.1.31

[B19] Veech RL The therapeutic implications of ketone bodies: the effects of ketone bodies in pathological conditions: ketosis, ketogenic diet, redox states, insulin resistance, and mitochondrial metabolism Prostaglandins Leukot Essent Fatty Acids 2004 70 309 319 10.1016/j.plefa.2003.09.007 14769489

[B20] Taggart AK Kero J Gan X Cai TQ Cheng K Ippolito M Ren N Kaplan R Wu K Wu TJ Jin L Liaw C Chen R Richman J Connolly D Offermanns S Wright SD Waters MG (D)-beta-Hydroxybutyrate inhibits adipocyte lipolysis via the nicotinic acid receptor PUMA-G J Biol Chem 2005 280 26649 26652 10.1074/jbc.C500213200 15929991

[B21] Kolovou GD Anagnostopoulou KK Daskalopoulou SS Mikhailidis DP Cokkinos DV Clinical relevance of postprandial lipaemia Curr Med Chem 2005 12 1931 1945 10.2174/0929867054546609 16101498

